# Essential Oils in Dentistry: Therapeutic Potential and Emerging Clinical Applications

**DOI:** 10.7759/cureus.105534

**Published:** 2026-03-19

**Authors:** Aysegul Sunar, Betül Buyukkilic Altinbasak

**Affiliations:** 1 Department of Oral Health and Dental Health, European Vocational School, Kocaeli Health and Technology University, Kocaeli, TUR; 2 Department of Pharmacognosy, Faculty of Pharmacy, Kocaeli Health and Technology University, Kocaeli, TUR

**Keywords:** aromatherapy, dentistry, essential oils, oral diseases, oral health

## Abstract

The escalating costs, side effects, and rising antimicrobial resistance associated with conventional dental therapies have intensified the search for plant-derived therapeutic agents. While there is a significant surge in academic research interest regarding the pharmacological potential of essential oils (EOs), their widespread integration into routine clinical dental practice remains an evolving process. For this reason, many traditional medicines are being reevaluated to determine their efficacy and safety profiles. This narrative review evaluates the current literature on EOs, focusing on their therapeutic potential and dental applications while distinguishing between promising laboratory findings and the current status of their practical adoption in dentistry. The review highlights that EOs may serve as adjunctive agents in protecting and maintaining oral health, provided that their clinical use is further standardized.

## Introduction and background

According to the World Oral Health Report, despite innovations in all areas, oral health problems continue to be observed globally, especially among impoverished groups [1]. Conversely, the World Health Organization notes that approximately 80% of the population utilizes herbal medicines, and the industrialization of these products is increasing significantly [2]. Essential oils (EOs), traditionally used as antioxidants in food preservation [3], are now recognized for possessing over 200 components with anticancer, antimicrobial, and anti-inflammatory properties [4,5]. Oral diseases can negatively impact overall health, altering an individual’s quality of life. Dental caries and periodontal diseases remain among the most significant global health issues [6].

Due to the limitations of standard therapeutic procedures, specifically rising bacterial resistance and high costs, the discovery of new therapeutic agents from herbal sources is gaining importance. Consequently, many drugs traditionally used to treat infections are being reevaluated through revisited clinical trials to determine their efficacy. In recent years, this has led to a burgeoning scholarly focus on EOs as a distinct category of natural therapeutic agents. However, it is essential to distinguish between this increasing research interest and established routine clinical use; while laboratory and preliminary clinical studies demonstrate significant potential, the widespread, standardized integration of EOs into daily dental practice is still limited and requires more robust clinical validation [7,8].

This review aims to evaluate the biological mechanisms and clinical potential of EOs as adjunctive therapeutic agents in modern dentistry.

## Review

Methods

Data Extraction and Synthesis

Data extraction focused on study design, types of EOs, and reported biological activities. The findings were synthesized narratively and organized according to major dental applications, such as oral hygiene, dental anxiety, and periodontology.

Literature Search Strategy

A narrative literature review was conducted to identify and evaluate studies investigating the use of EOs in dentistry and oral health care. The literature search covered publications from January 1995 to December 2025. The following electronic databases were systematically searched: PubMed, Scopus, Web of Science, and Google Scholar.

The search strategy was designed to capture studies related to both aromatherapy and dental sciences. Primary keywords and search strings included combinations of the following terms: “essential oils”, “aromatherapy”, “dentistry”, “oral health”, “dental anxiety”, “periodontal diseases”, “endodontics”, “oral microbiota”, and “biofilm”. Boolean operators (AND, OR) were used to refine the search strategy (e.g., “essential oils AND dentistry”, “aromatherapy AND dental anxiety”).

Eligibility Criteria

Studies were considered eligible for inclusion if they met the following criteria: (i) original in vitro, in vivo, or clinical studies; (ii) review articles addressing dental or oral health-related applications of EOs; (iii) studies evaluating antimicrobial, anti-inflammatory, anxiolytic, wound healing, or antibiofilm effects of EOs within an oral or dental context; and (iv) articles published in English. Exclusion criteria included non-peer-reviewed publications, conference abstracts without full text, studies unrelated to dentistry or oral health, non-English articles, and studies lacking sufficient methodological detail to ensure data reliability.

Study Selection Process

Initially, 175 records were identified through database searches, including a targeted supplementary search to incorporate foundational and recent evidence on turmeric EO. After removing duplicates and screening titles and abstracts, 93 studies were excluded because they were either not directly related to dentistry, lacked phytochemical detail, or were non-English publications. Consequently, 73 full-text articles met the inclusion criteria and were included in the qualitative synthesis of this review.

Methodological Considerations

Given the predominance of in vitro studies and the variability in experimental protocols, the findings were interpreted with particular attention to their translational relevance. Differences between in vitro results and potential in vivo or clinical outcomes were critically evaluated. Furthermore, limitations within the current literature were highlighted, and existing gaps requiring further investigation were identified.

EOs

To date, approximately 3,000 EOs have been identified [9]. Beyond their applications in cosmetics and religious practices, they have a long history of medicinal use, dating back to Ancient Egypt around 3500 BCE, where they were administered as ointments, inhalations, powders, pills, and macerated extracts [10]. Since antiquity, EOs have been incorporated into various traditional medical and dental practices. These substances are plant-derived secondary metabolites known for their antibacterial, antifungal, and antioxidant activities [11-13]. Interest in aromatherapy increased notably during the 1980s, in parallel with advances in mind-body medicine and psychoneuroimmunology, and it is now well recognized that specific olfactory stimuli may influence emotional and psychological states [14,15].

EOs are synthesized in different plant organs, including leaves, flowers, fruits, seeds, resins, bark, and wood, and consist of volatile aromatic compounds responsible for the characteristic scent and flavor of the plant. The term “essential” refers to their ability to represent the plant’s essence rather than their chemical nature. Although described as oils, they are generally less viscous and more volatile than fixed oils and are also referred to as ethereal or volatile oils [16,17].

Chemically, EOs are primarily composed of hydrocarbon terpenes and terpenoids [18-20], accompanied by alcohols, aldehydes, ketones, esters, acids, epoxides, amines, sulfides, oxides, fatty acids, and other sulfur-containing derivatives. Key bioactive constituents include terpineol, thujanol, myrcenol, neral, thujone, camphor, and carvone [21,22]. Monoterpenes and sesquiterpenes constitute the majority of terpenoid compounds, while oxygenated derivatives form an additional subgroup [19]. Due to their broad therapeutic potential, monoterpenes have been extensively investigated [23]. The antimicrobial properties of EOs may be bactericidal or bacteriostatic and are influenced by chemical composition, molecular structure, concentration, and synergistic interactions among constituents [24-26].

A defining characteristic of EOs is their pronounced compositional variability. Oils obtained from the same plant species may differ depending on geographical origin, plant maturity, genetic factors, bacterial endophytes, and extraction techniques [27-29]. Such variability contributes to differences in biological activity. The mechanisms of action of EOs are closely related to their chemical composition and functional groups, with membrane solubility playing a critical role in antimicrobial efficacy through disruption of phospholipid bilayers [30,31].

Historical documentation of EO production is often attributed to Ibn al-Baytar (1188-1248), who described early extraction methods [29]. EOs are complex mixtures of volatile compounds, primarily consisting of terpenoids and phenolic derivatives [32,33]. Unlike crude plant extracts, which may contain nonvolatile phytochemicals such as alkaloids, flavonoids, and glycosides, EOs are characterized by their low molecular weight and hydrophobic nature. Extraction methods are adapted to these properties and include both traditional and advanced techniques. Contemporary approaches are increasingly favored due to reduced extraction time, lower energy and solvent consumption, and decreased environmental impact [5]. Common methods include hydrodistillation, Clevenger hydrodistillation, and microwave-assisted hydrodistillation, with the latter demonstrating significantly higher efficiency [34,35]. Additional techniques such as hydrolysis, grinding, fermentation, and solvent extraction have also been described [36].

Oral health reflects the structural and functional integrity of the teeth, gingiva, tongue, oral mucosa, and the entire orofacial system. The most prevalent oral diseases include dental caries, gingivitis, periodontitis, and oral cancers. Within dentistry, EOs have been investigated for a wide range of applications. Clove oil, commonly referred to as eugenol, has been extensively used in endodontic practice due to its analgesic and antiseptic properties. Other EOs, including tea tree, thyme, cinnamon, citrus, bergamot, lavender, and peppermint oils, are currently under investigation for their therapeutic potential [37].

In the management of dental caries, EOs such as clove, sesame, cinnamon, sumac, and citrus oils have demonstrated antibacterial, antifungal, anticariogenic, and anti-adhesive effects [17,38-40]. For periodontal conditions, clove, lavender, lemongrass, and eucalyptus oils have been evaluated for their anti-inflammatory and antibiofilm activities [17,36,41]. Lavender and clove oils have also been studied for dental pain control due to their anxiolytic, analgesic, and anti-inflammatory properties [15,17,42]. In oral oncology, clove and cinnamon oils have been investigated for anti-inflammatory, antimutagenic, cytotoxic, and immunomodulatory effects [43-46]. In addition, EOs may contribute to improved patient comfort by reducing anxiety, depression, and nausea associated with dental procedures [47,48].

Antimicrobial effects have been reported against *Streptococcus mutans*, *Streptococcus sobrinus*, *Streptococcus salivarius*, *Streptococcus sanguinis*, and *Lactobacillus acidophilus *[48]. Antifungal effects against *Candida albicans* have also been reported, with oregano oil shown to inhibit adhesion and biofilm formation [42,49,50]. These antimicrobial effects are primarily attributed to increased cell membrane permeability, leading to leakage of intracellular components and cell lysis, as well as interference with cellular metabolism and induction of oxidative stress [51,52].

Use of EOs as Products in Dentistry

Despite growing interest, the application of EOs in dentistry remains insufficiently explored. Existing studies indicate promising outcomes; however, their clinical use has not yet been fully standardized. Currently, EOs are incorporated into dental products such as sprays, mouthwashes, gargles, gels, and toothpastes and are utilized across multiple dental disciplines, including endodontics, pediatric dentistry, oral surgery, periodontology, and general oral health care [53,54].

Effects of EOs on Oral Hygiene

Since the 19th century, EOs have been included in mouthwash formulations for the prevention of dental diseases. Previous reports indicate that EO-based mouth rinses can reduce salivary bacterial counts by approximately 10-20%, with effects persisting for several hours after use [53]. Randomized clinical studies have shown that such formulations may be more effective in reducing plaque accumulation, gingivitis, and periodontitis than mouthwashes containing low concentrations of cetylpyridinium chloride [54]. Although short-term daily use is generally well tolerated, some studies have reported varying degrees of cytotoxicity, emphasizing the importance of concentration-dependent evaluation [55].

Chlorhexidine (CHX) remains the gold standard for plaque control and the treatment of gingivitis and periodontitis; however, its long-term use is associated with adverse effects such as tooth discoloration, taste alteration, mucosal desquamation, and supragingival calculus formation. In this context, EOs are considered a viable alternative to CHX-based products [54,55].

Lavender oil has demonstrated antibacterial activity and is commonly used for infections of the oral cavity and upper respiratory tract [56]. Oregano oil exhibits antiviral activity against herpes simplex virus in addition to bacteriostatic effects [36,56]. Citrus-derived EOs, including sweet orange, bergamot, lemon, lime, grapefruit, and kumquat, have been investigated for use in mouthwash formulations due to their antibacterial, antifungal, antioxidant, anti-inflammatory, and anticancer properties, although data specific to oral pathology remain limited [57,58]. Despite the potential of natural-based formulations, CHX-containing mouthwashes continue to represent the clinical reference standard [59].

Use of EOs in dentistry

EOs as Anxiolytics in Dentistry

Aromatherapy is classified as a complementary therapeutic approach and involves the administration of EOs through inhalation, transdermal absorption, or oral routes for preventive and supportive medical care. In recent years, aromatherapy has been increasingly applied for the management of anxiety, insomnia, depressive symptoms, and cognitive disorders. Accumulating evidence suggests that EOs may exert measurable pharmacological effects while being associated with fewer adverse reactions than conventional psychotropic medications [59].

One commonly used aromatherapeutic method involves dispersing volatile EO compounds into the surrounding environment. Upon inhalation, aromatic molecules interact with receptors in the nasal mucosa and are transmitted via neural pathways to the brain, particularly to limbic structures involved in emotional processing. These signals may induce physiological and psychological responses that influence mood, stress levels, and cognitive function [60,61].

Dental anxiety is a frequent clinical concern that can negatively affect patient cooperation and treatment outcomes. In dental settings, aromatherapy has been investigated as a noninvasive approach to stress management. Studies conducted in waiting areas have shown that exposure to lavender or citrus-derived EOs may reduce salivary cortisol levels and heart rate, indicating decreased stress and anxiety. These physiological changes are clinically significant as they often translate into enhanced patient cooperation, allowing for smoother clinical procedures and potentially shorter treatment times. By stabilizing the patient’s autonomic response before entering the operatory, aromatherapy helps mitigate the “white coat effect,” thereby improving the overall quality of the dental experience and long-term treatment outcomes [61,62]. Furthermore, the use of diluted lavender oil via candle warmers or diffusers prior to dental procedures has been associated with enhanced sedation, improved mood, and reduced anxiety [61]. Kim et al. additionally reported that lavender oil exposure was associated with a reduced perception of injection-related pain during dental treatment [62].

In a study by Cai et al., aromatherapy applied in dental clinics was found to be more effective in reducing dental anxiety compared with negative control groups without aromatherapy, with outcomes comparable to those achieved using music-based interventions. Among the EOs evaluated, lavender was the most frequently used, followed by orange oil. The authors emphasized the need for further clinical studies to establish standardized aromatherapy protocols in dental practice [63].

Use of EOs for Wound Healing in Dentistry

EO-enriched wound care products have gained attention for their potential applications in dentistry and oral surgery. These formulations commonly contain oils such as tea tree, lavender, and eucalyptus, which are recognized for their antimicrobial and anti-inflammatory properties and may contribute to enhanced wound healing and reduced postoperative infection risk [64].

Wang et al. reported that wound ointments containing EOs provided improved therapeutic outcomes and facilitated healing following oral surgical procedures [65]. In implant dentistry, wound dressings incorporating EOs have demonstrated antibiofilm activity and have been proposed as alternatives to methylparaben-containing products in patients with allergic sensitivity. However, further clinical trials are required to confirm safety and long-term efficacy.

Gheorghita et al. demonstrated that fennel, mint, pine, and thyme oils exhibit significant antimicrobial activity against oral and opportunistic pathogens, including *Staphylococcus aureus*, *Enterococcus faecalis*, *Escherichia coli*, *Pseudomonas aeruginosa*, and *C. albicans *[64]. These findings support the potential use of EOs as adjunctive agents in wound management following dental surgical interventions.

Endodontics and EOs

The removal of microorganisms from root canals is a crucial criterion for successful endodontic treatment. If microorganisms are not effectively eradicated, they can lead to resistant infections and poor healing. *E. faecalis *is commonly found in root canals diagnosed with apical periodontitis and is a primary pathogen in secondary endodontic infections. This bacterium can survive in harsh, nutrient-deficient environments and grow by forming a biofilm on root canal walls. Cosan et al. demonstrated that a material combined with calcium hydroxide, *Mentha spicata*, and *Origanum dubium *exhibited significant antimicrobial activity and was successfully used in root canal instrumentation, irrigation, and canal disinfection [66].

Marinković et al. utilized a product containing* Cymbopogon martinii *and *Thymus zygis *in the root canals of extracted teeth, which demonstrated antimicrobial activity [67]. Additionally, new resin-based sealers incorporating natural oils have shown potential as canal materials in endodontics, compared with commonly used commercial sealers, due to their favorable physical and chemical properties, antimicrobial effects, and biocompatibility [68].

Prosthetics and EOs

In prosthodontics, EOs have been incorporated into restorative and supportive materials due to their antimicrobial properties. An EO-based glass ionomer cement has been shown to inhibit the growth of *S. mutans *and *C. albicans*, microorganisms commonly associated with prosthesis-related oral infections. Clinical observations indicate that patients with prosthetic stomatitis experienced partial or complete symptom resolution following the use of gels containing EOs. After six months of application, these formulations were reported to be as effective as CHX in controlling gingival inflammation, highlighting their potential as adjunctive agents in prosthetic care [69].

Periodontology and EOs

Periodontal diseases are closely associated with microbial biofilm formation and host inflammatory responses. EOs have been investigated for their capacity to modulate both microbial load and inflammation within periodontal tissues. Eucalyptus oil, in particular, has demonstrated antibacterial effects by reducing plaque activity against *Porphyromonas gingivalis *and *S. mutans*, key pathogens implicated in periodontitis and other oral diseases. In addition to aromatic plant oils, coconut oil has been evaluated for its potential role in the management of gingivitis and periodontitis due to its biological activity, low cost, and favorable safety profile [70]. Mostafa et al. reported improvements in gingival and periodontal tissue health following the application of sesame and coconut oils using a dermaroller technique. The authors suggested that interactions between salivary alkaline components and the applied oils contributed to reduced plaque adhesion and inflammation [71].

Overall, the use of EOs within safe concentration ranges appears to play a supportive role in inhibiting biofilm formation through antimicrobial mechanisms, thereby suggesting potential benefits in the prevention and management of gingivitis and periodontitis [72,73].

Limitations

The findings of this review should be interpreted with caution due to several limitations. First, most available evidence on the use of EOs in dentistry is derived from in vitro studies, which may not fully represent clinical conditions, as factors such as salivary flow, protein interactions, and oral biofilm complexity can influence biological activity in vivo. Consequently, as emphasized in recent dental literature, there is a critical requirement for more comprehensive clinical trials to validate the safety and therapeutic efficacy of these oils before they can be fully integrated into standard clinical practice.

Second, substantial heterogeneity exists among studies with respect to EO composition, concentration, extraction methods, and experimental design, limiting direct comparability and contributing to variability in reported outcomes. Variability in chemical composition resulting from botanical origin, harvesting conditions, and extraction methods adds to this heterogeneity. In addition, potential dose-dependent cytotoxicity and allergic hypersensitivity must be considered; however, available evidence suggests that maintaining agents at relatively low concentrations is associated with a favorable safety profile in human oral cells.

Finally, clinical evidence remains limited, with relatively few randomized controlled trials available to establish efficacy, optimal dosing, and long-term safety. As this review was conducted using a narrative approach rather than a systematic methodology, there is an inherent risk of selection bias, and no quantitative synthesis was performed. While this approach allows for a broad and integrative discussion, it does not provide the level of evidence grading associated with systematic reviews or meta-analyses.

Future directions

Future research should prioritize well-designed randomized controlled trials to evaluate the in vivo efficacy, safety profiles, and minimum effective concentrations of EOs in dental applications. Standardization of EO composition, along with detailed chemical characterization, is essential to reduce variability and improve reproducibility across studies. Additionally, investigations into patient acceptance, particularly regarding sensory preferences and tolerability, are necessary to optimize formulation strategies. Advances in nanotechnology and targeted delivery systems offer promising opportunities to enhance the stability, bioavailability, and antimicrobial performance of EO-derived bioactive compounds. Collectively, these efforts will be critical for translating traditional and experimental evidence into clinically applicable, standardized dental therapies.

## Conclusions

EOs have gained attention as complementary agents in dentistry due to their reported antimicrobial, anti-inflammatory, anxiolytic, and biofilm-inhibitory properties. Evidence from in vitro and limited in vivo studies indicates that certain EOs can act against oral pathogens, including those associated with halitosis, sometimes showing efficacy comparable to CHX. EOs should be considered strictly as adjunctive supportive agents rather than alternatives to established evidence-based therapies. Future robust, long-term clinical trials are essential to confirm their safety, efficacy, and optimal application in routine dental practice.
